# Enteral Immunomodulatory Diet (Omega-3 Fatty Acid, γ-Linolenic Acid and Antioxidant Supplementation) for Acute Lung Injury and Acute Respiratory Distress Syndrome: An Updated Systematic Review and Meta-Analysis

**DOI:** 10.3390/nu7075239

**Published:** 2015-07-09

**Authors:** Congcong Li, Liyan Bo, Wei Liu, Xi Lu, Faguang Jin

**Affiliations:** Department of Respiratory and Critical Care Medicine, Tangdu Hospital, Fourth Military Medical University, Xinsi Road 1, Xi’an 710038, China; E-Mails: licong1988@hotmail.com (C.L.); boliyan@hotmail.com (L.B.); liuweilung@163.com (W.L.)

**Keywords:** enteral nutrition, immunomodulatory diet, acute respiratory distress syndrome, acute lung injury, critical care, mortality

## Abstract

Enteral immunomodulatory nutrition is considered as a promising therapy for the treatment of acute lung injury and acute respiratory distress syndrome (ALI/ARDS). However, there are still some divergences, and it is unclear whether this treatment should be recommended for patients with ALI/ARDS. Therefore, we conducted this systematic review and meta-analysis to assess the efficacy and safety of an enteral immunomodulatory diet on the clinical outcomes of ALI/ARDS patients. Methods: We retrieved potentially relevant clinical trials though electronic databases. All trials of enteral immunomodulatory diet for ALI/ARDS were included. Analyses of the overall all-cause mortality, 28-day ventilator-free days and 28-day intensive care unit (ICU) free days were conducted. Results: In total six controlled trials were evaluated. The pooled results did not show a significant reduction in the risk of all-cause mortality (M-H RR (the overall Mantel-Haenszel relative risk), 0.81 (95% CI, 0.50–1.31); *p* = 0.38; 6 trials, *n* = 717) in ALI/ARDS patients treated with the immunomodulatory diet. This treatment also did not extend the ventilator-free days and ICU-free days. However, patients with high mortality might benefit from this treatment. Conclusions: The enteral immunomodulatory diet could not reduce the severity of the patients with ALI/ARDS. Whereas, for ALI/ARDS patients with high mortality, this treatment might reduce the all-cause mortality, but its use should be treated with discretion.

## 1. Introduction

Since its first description in 1967, acute lung injury (ALI) and acute respiratory distress syndrome (ARDS) have been known as common and lethal diseases. With mortality ranging from 25%–40% [1], ALI/ARDS is a life-threatening disorder that cannot be ignored. It is mainly caused by predisposing disorders such as pneumonia, aspiration, shock, and severe sepsis [2]. Benefiting from the exploration of the pathophysiology of ALI/ARDS, we know that after having been affected by these diseases, neutrophils will infiltrate into the alveolar space and pulmonary mesenchyme, where they will release pro-inflammatory cytokines and eventually cause ALI/ARDS [2], which is characteristic of leakage of edema fluid and mismatch of ventilation and perfusion [2,3].

Although we know much about the pathophysiologic change of ALI/ARDS, very little improvement in patient outcomes has been achieved. The main treatment is supportive care, including maintaining oxygenation and avoiding complications [1,2]. There are no specific and effective treatments for ALI/ARDS [4], although many ventilation strategies and medicines have been tried. Thus, it is urgent to find an effective treatment for ALI/ARDS. Over the past two decades, some trials [5,6,7] and meta-analyses [8,9] have suggested that the enteral use of an immunomodulatory diet (omega-3 fatty acid, γ-linolenic acid and antioxidant supplementation) might be a promising therapy.

This immunomodulatory diet is mainly combined with anti-inflammatory elements (such as eicosapentaenoic acid (EPA), docosahexaenoic acid (DHA) and gamma-linolenic acid (GLA)) and antioxidants (such as vitamin C, vitamin E and beta-carotene). It has been reported that Omega-3 (EPA and DHA) could modulate inflammatory processes, such as by reducing leukotriene production [10,11] and decreasing the synthesis of prostaglandin E2 [12]. It can also reduce the permeability of the alveolar-capillary membrane [13]. As for the antioxidants, they can scavenge free radicals, as we all know, and thus reduce the inflammation [14].

Using enteral nutrition for ALI/ARDS patients has been demonstrated to improve oxygenation and extend 28-day ventilator-free days and 28-day intensive care unit (ICU) free days [5,7]. It has even been associated with reduced mortality [6,7]. Some meta-analyses [8,9] have also shown its effect. However, one trial conducted by Rice *et al.* [15] revealed that an enteral inflammation-modulating diet did not improve the outcomes of ALI/ARDS patients and might be harmful. This conclusion compelled us to re-evaluate the effectiveness and safety of this treatment.

Therefore, we conducted this systematic review and meta-analysis to re-evaluate the effectiveness and safety of enteral use of the immunomodulatory diet (omega-3 fatty acid, γ-linolenic acid and antioxidant supplementation) *vs.* standard enteral nutrition on the mortality and clinical outcomes in patients with ALI/ARDS and to guide further research in this area.

## 2. Methods

The work, including the literature search, study selection and data extraction, was conducted according to standard strategies described below. Two reviewers (CCL and LYB) completed this work independently, and all discrepancies were solved by discussion or consultation with the senior reviewer (FGJ). Ethical approval was not required to conduct this meta-analysis.

### 2.1. Search Strategy

An extensive computer search of the relevant literature was performed by the two reviewers independently using databases including MEDLINE (PubMed), Embase and the Cochrane Central Register of Controlled Trials. We also retrieved potentially relevant literature manually, including conference abstracts published in the American Journal of Respiratory and Critical Care Medicine, Critical Care Medicine and Chest. All articles and conference abstracts about enteral nutrition therapies for patients with ALI or ARDS were identified regardless of language. The search terms we used were critically ill patients, acute lung injury, ALI, acute respiratory distress syndrome, ARDS, mechanical ventilation, sepsis, immunomodulatory diet, fish oil, antioxidants, omega-3 fatty acids, eicosapentaenoic acid (EPA), docosahexaenoic acid (DHA) and γ-linolenic acid (GLA).

### 2.2. Study Selection

Studies were included if they fulfilled all of the inclusion criteria. (1) Participants: patients had to be diagnosed with ALI/ARDS or have respiratory failure that required mechanical ventilation. (2) Type of studies: studies were eligible only if they were randomized controlled trials. (3) Type of interventions: studies used enteral nutrition therapies (omega-3 fatty acids, γ-linolenic acid and antioxidants). Studies were excluded if they did not provide outcomes related to mortality, 28-day ventilator-free days or 28-day ICU-free days. Crossover studies were also excluded.

### 2.3. End Points and Data Extraction

The primary end point was all-cause mortality, and the secondary end points were 28-day ventilator-free days, 28-day ICU-free days and adverse effects. For all-cause mortality, we used 28-day mortality. If 28-day mortality could not be acquired, we used ICU or hospital mortality instead. We also extracted and collected the relevant information about each study, such as the characteristics of the studies, characteristics of the participants, enteral immunomodulatory therapy strategies and types of outcomes.

### 2.4. Quality Assessment

The quality levels of the included trials were also evaluated independently by two authors (CCL and LYB). We assessed the risk of bias (including selection bias, performance bias, attrition bias, detection bias, reporting bias and other bias) using the assessment table recommended by the Cochrane Reviewers’ Handbook [16]. We also evaluated the methodological quality of the included trials using the Modified Jadad Scale [17], where the full score is 7, and scores of 4–7 are regarded as high quality and 1–3 as low quality.

### 2.5. Data Processing and Statistical Analysis

First, we examine the heterogeneity of the included studies using the *I*^2^ statistic and Chi^2^ test, with significant heterogeneity if *p* ≤ 0.10 for the Chi^2^ test or *I*^2^ ≥ 50%. If significant heterogeneity was obtained, we would use the random-effects model for the following analysis; otherwise, the fixed-effects model would be used.

Second, we pooled the treatment effects of enteral nutrition on the all-cause mortality to estimate the summary effect. As the mortality outcome was dichotomous, we calculated the relative risk (RR) and 95% confidence interval (CI) of every included trial and then pooled them to estimate the overall Mantel-Haenszel (M-H) RR and the 95% CI. For the continuous variables, we calculated the standardized mean difference (SMD). To test the robustness of the results, we performed a sensitivity analysis by excluding each individual study and re-analyzing. The funnel plot was calculated to evaluate the publication bias.

The results were considered statistically significant if (1) the two-sided *p*-value ≤ 0.05, (2) the confidence interval for RR did not include 1, and (3) the confidence interval for SMD did not include 0. The data synthesis and sensitivity analyses were performed using Review Manager (version 5.1).

## 3. Results

### 3.1. Study Selection and Quality Assessment

**Figure 1:**
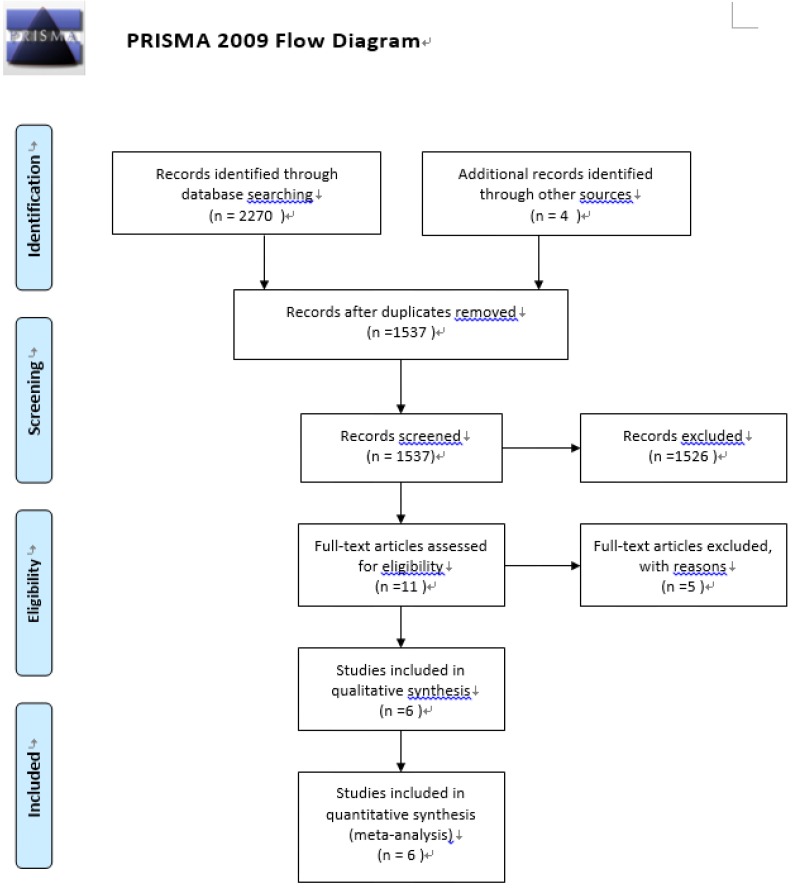
Preferred Reporting Items for Systematic Reviews and Meta-Analyses (PRISMA) Flow Diagram.

We identified six studies [5,6,7,15,18,19] that fulfilled our inclusion criteria out of 2274 potential articles though searching the relevant databases (see Figure 1). All of them were included in our analysis. Five relevant papers [20,21,22,23,24] were excluded based on the reasons described in Table S1. The major characteristics of the six included trials are summarized in Table 1. In short, the trials encompassed a total of 717 patients, with 365 patients in the experimental groups and 352 patients in the control groups. The mean age of the patients ranged from 51.0 to 65.1. The mortality of the control groups ranged from 12.5% to 57.14%. When stratified by the compositions of the immunomodulatory diet, two studies included treatment with EPA + GLA + antioxidants, and four studies included treatment with EPA + DHA + GLA + antioxidants. When stratified by the blind strategies, four trials were double-blind, one trial was single-blind and one trial was unblinded.

We evaluated the quality of the included trials using the Modified Jadad Scale and Cochrane’s risk of bias assessment table. As shown in Table S2, all of the included studies were high quality, and most of them had low risk of bias in the generation of random sequence, allocation concealment, incomplete outcome data and selective reporting. Only two trials were high risk in terms of the blinding of participants and personnel.

**Table 1:** Characteristics of Included Trials.

Parameter	Gadek *et al*., 1999	Singe *et al*., 2006	Pontes-Arda *et al.*, 2006	Grau-Carmona *et al.*, 2011	Rice *et al.*, 2011	Elamin *et al.*, 2012
Interventions	EPA + GLA + antioxidants	EPA + GLA + antioxidants	EPA + DHA + GLA + antioxidants	EPA + DHA + GLA + antioxidants	EPA + DHA + GLA + antioxidants	EPA + DHA + GLA + antioxidants
Control Diet	Isonitrogenous andisocaloric control diet	Isonitrogenous and isocaloric control diet	Isonitrogenous and isocaloric control diet	Isocaloric control diet	Isocaloric and isovolemic control diet	Isonitrogenous and isocaloric control diet
Treatment Duration	N/A	14 days	N/A	N/A	21 days	7 days
Route	Gastric, duodenal, jejunalfeeding tube	Nasogastric, duodenal, jejunal tube	Eneral feeding	Gastric, jejunal tube	Bolus delivery	Nasogastric, nasoduodenal, nasojejunal, jejunostomytubes
Sample Size						
Treatment Group	51	46	55	61	143	9
Control Group	47	49	48	71	129	8
Sex Ratio (Male:Female)	52:46	NA	61:42	30:132	133:139	8:9
Average Age (years)	51	59.7	65.1	63	54.1	52.4
No. of Participants Drop-out or Withdrawal	48	5	62	28	0	5
Blind Type	Double-blind	Unblind	Double-blind	Single-blind	Double-blind	Double-blind
Mordified Jadad Scale	7	5	5	5	7	5
Primary End Point	Time receiving ventilatorysupport	Change in oxygenation and breathing patterns	28-day mortality	New organ dysfunction	Ventilator-free days	Oxygenation and modified Lung Injury Scores
Mortality Outcome Type	Mortality	28-day mortality	28-day mortality	28-day mortality	60-day or hospital mortality	28-day mortality
Mortality						
Treatment Group	6/51	13/46	18/55	11/61	38/143	0/9
Control Group	9/47	28/49	25/48	11/71	21/129	1/8
Mortality Rate of Control Group	9/47 (19.15%)	28/49 (57.14%)	25/48 (52.08)	11/71 (15.49)	21/129 (16.28)	1/8 (12.5)
PaO_2_/FiO_2_ Ratio (Day 7)						
Treatment Group	N/A	296.5 ± 165.3 (SD)	224.4	217	N/A	178
Control Group	N/A	236.3 ± 79.8 (SD)	150.5	190	N/A	201

Abbreviations: EPA, eicosapentaenoic acid; GLA, gamma-linolenic acid; DHA, docosahexaenoic acid; N/A, not available.

### 3.2. Effect on Mortality

**Figure 2:**
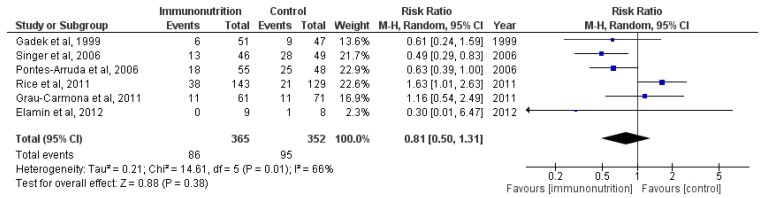
Forest plot of the association between enteral immunomodulatory diet and all-cause mortality among patients with ALI (acute lung injury)/ARDS (acute respiratory distress syndrome).

Because significant heterogeneity was found across the included trials (*χ*^2^ = 14.61, df = 5 (*p* = 0.01); *I*^2^ = 66%), we used the random-effects model to analyze the overall effect of immunomodulatory nutrition on mortality. As shown in Figure 2, there was no significant difference between the two groups (M-H RR, 0.81 (95% CI, 0.50–1.31); *p* = 0.38; six trials, *n* = 717) that is, the pooled result did not showed a significant reduction in the risk of all-cause mortality in ALI/ARDS patients treated with immunomodulatory nutrition. The overall mortality of the six trials was 25.24%, and the mortality of the experimental groups was 23.56% compared with 26.99% for the control groups.

**Figure 3:**
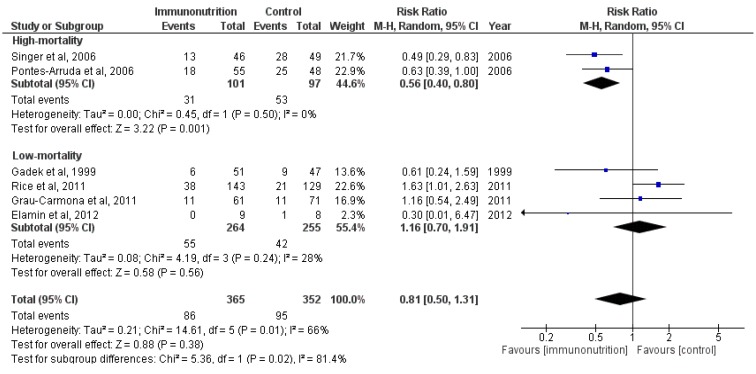
Forest plot of the association between enteral immunomodulatory diet and all-cause mortality among patients with ALI/ARDS, stratified by discrepancy of mortality.

Because of the heterogeneity of the mortality in the control groups across the included trials, we conducted a subgroup analysis by stratifying the previous meta-analyses according to the mortality of the control groups. The analyses (M-H RR, 1.16 (95% CI, 0.70–1.91); *p* = 0.56; three trials, *n* = 97) revealed that for patients with low mortality, this treatment could not reduce the overall mortality in ALI/ARDS patients (see Figure 3). The results (M-H RR, 0.56 (95% CI, 0.40–0.80); *p* = 0.001; two trials, *n* = 198) indicated that patients with high mortality might benefit from this treatment, and there was a significant subgroup difference (*χ*^2^ = 5.36, df = 1 (*p* = 0.02); *I*^2^ = 81.4%). However, they were something that need our attention. The quality of the trials in this subgroup was lower than most of others (as shown in Table S2).

### 3.3. Effect on 28-Day Ventilator-Free Days and 28-Day ICU-Free Days

**Figure 4:**
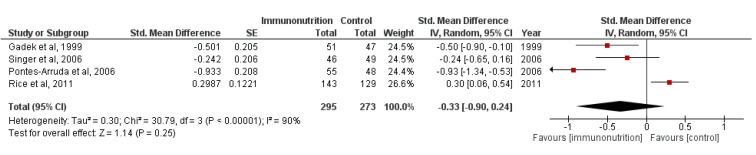
Forest plot of the association between enteral immunomodulatory diet and 28-day ventilator-free days among patients with ALI/ARDS.

**Figure 5:**
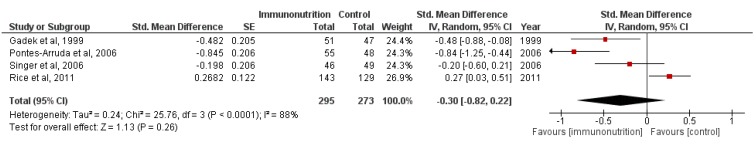
Forest plot of the association between enteral immunomodulatory diet and 28-day ICU-free days among patients with ALI/ARDS.

We also pooled the data about the 28-day ventilator-free days and 28-day ICU-free days. The outcomes of 568 participants from four trials were available when assessing the effect of enteral nutrition on ventilator-free days and ICU-free days. As shown in Figure 4 and Figure 5, enteral nutrition did not extend the ventilator-free days (M-H RR, −0.33 (95% CI, −0.90–0.24); *p* = 0.25; four trials, *n* = 568) and ICU-free days (M-H RR, −0.30 (95% CI, −0.82–0.22); *p* = 0.26; four trials, *n* = 568). Because of the significant heterogeneity of the included trials ((*χ*^2^ = 30.79, df = 3 (*p* < 0.00001); *I*^2^ = 90%) and (*χ*^2^ = 25.76, df = 3 (*p* < 0.0001); *I*^2^ = 88%)), the random-effects model was selected.

### 3.4. Sensitivity Analyses

To test the robustness of the results, we conducted sensitivity analyses. We excluded each individual study, re-analyzing and comparing with the original results. When excluding the trial conducted by Rice T. *et al.* [15], the overall effect was M-H RR, 0.63 (95% CI, 0.47–0.85); *p* = 0.0.003; five trials, *n* = 445 (see Figure S1). When excluding other trials, the results were consistent with the previous one.

### 3.5. Adverse Effects

**Figure 6:**
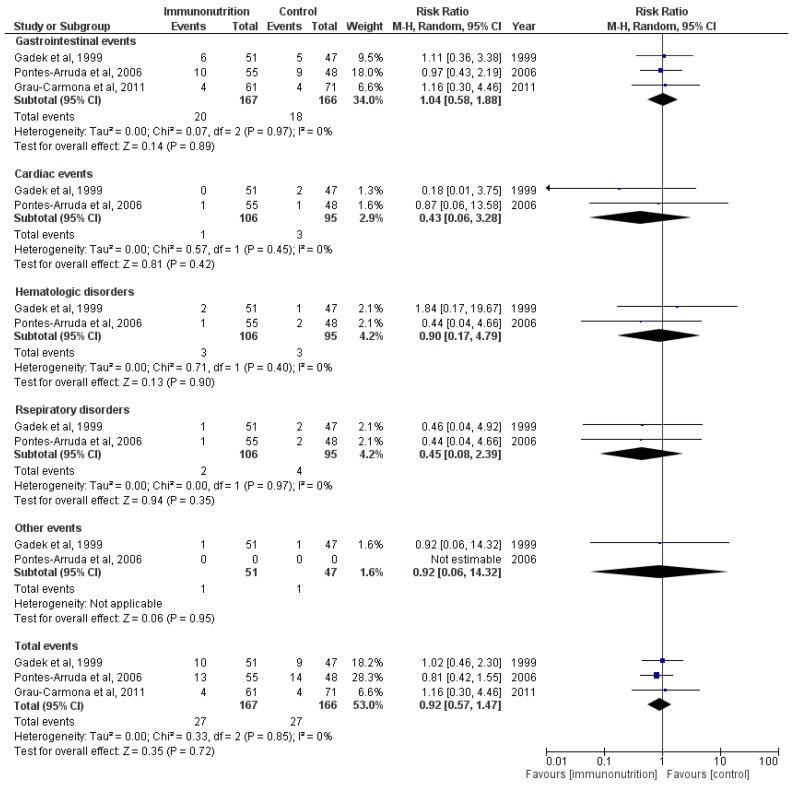
Forest plot of the association between enteral immunomodulatory diet and adverse events among patients with ALI/ARDS.

To test the safety of this treatment, we also analyzed the adverse effects of the enteral immunomodulatory diet. The majority of adverse events were gastrointestinal events such as diarrhea, dyspepsia and nausea. As shown in Figure 6, there was no significant difference between the two groups (M-H RR, 0.92 (95% CI, 0.57–1.47); *p* =0.72; three trials, *n* =333).

### 3.6. Publication Bias

No evidence of publication bias was detected by funnel plots (see Figure S2).

## 4. Discussion

In conducting this systematic review, we searched the relevant literature comprehensively without language limitation. The pooled results from all six independently conducted trials revealed that an enteral immunomodulatory diet (omega-3 fatty acid, γ-linolenic acid and antioxidant supplementation) could not improve all-cause mortality, ventilator-free days or ICU-free days in patients with ALI/ARDS. Overall, patients could not benefit from enteral immunomodulatory diet, and its use should be treated with discretion.

It was believed previously that the immunomodulatory diet could suppress the elevated inflammatory reactions during ALI/ARDS [5], and patients could benefit from it [6]. Preclinical studies reported that Omega-3 (EPA and DHA) could reduce leukotriene synthesis and the production of prostaglandin E2, which could be beneficial in ALI/ARDS [3,11,13]. The antioxidants could also reduce the inflammation through scavenging free radicals [25]. Several clinical trials confirmed these results [5,6,7], and demonstrated an association between the usage of enteral immunomodulatory diet and improved outcomes in ALI/ARDS patients [5,6,7]. Two meta-analyses [8,9] also demonstrated this effect. However, some trials conducted recently achieved a contrary result [15,18], showing that enteral inflammation-modulating diet did not improve the outcomes and might be harmful. Our results were similar. However, some results needed extra attention. As shown in the characteristics of the included studies, the mortality of the control groups varied widely (from 12.50% to 57.14%), and the test for heterogeneity was also significant for mortality. This result may be due to the different severity of the illness and improved treatment strategies [2]. To decrease its influence on the final results, we used the random-effects model for analysis, and we also conducted a subgroup analysis stratified according to the mortality of the control groups. The result revealed that enteral immunomodulatory nutrition could only benefit ALI/ARDS patients with high mortality. For patients with low mortality, this treatment had no effect and might be harmful. From this perspective, it is important to clarify the indications of this treatment, and for future trials about this aspect, the enrolled patients could be restricted to severe cases. However, the quality of the two trials included in the high-mortality subgroup was lower than most of the others, and the results of these studies might be affected.

The drop-out proportions of most included studies were large. Undoubtedly, the reliabilities of the final results achieved by these trials were influenced by this factor [16]. The main reason that people left the studies was that the patients could not tolerate the rate of continuous enteral infusions because of gastrointestinal complications [5,7]. However, the study conducted by Rice T. *et al.* [15] solved this problem by using bolus delivery, namely small-volume supplementation, to deliver the supplements. The results indicated that this method was more tolerable. However, given 120 mL fluid once might increase the risk of aspiration, especially for patients who already have respiratory compromise.

In this review, we demonstrated that ALI/ARDS patients could not benefit from enteral immunomodulatory diet through including some newly reported trials. However, we still need further exploration of the following issues. During sensitivity analyses, we found that the results were not very robust. The final conclusion was seriously affected by the trial conducted by Rice T. *et al*. When we excluded this study, re-analyzed and compared with the previous results, the opposite conclusion was obtained. This condition was more or less due to the discrepancy of the controlled nutrition, and the calorie intake was quite low in Rice T. *et al*.’s trials [26]. However, the reason is still unclear, and we should be aware that the conclusion is not certain. Further improved randomized clinical trials are needed.

Some limitations in this report should be mentioned. First, the heterogeneity tests of the all-cause mortality, ventilator-free days and ICU-free days were positive. Although we tried to reduce their influence methodologically (using a random-effects model and subgroup analyses), they might still cause some biases. Second, the sample sizes of the included trials were small, and only three trials had more than 100 patients available. Even worse, the drop-out proportions were large in the majority of the included trials. Third, there was also some variability in the patient types, outcome types, and route of intervention administration. When trying to solve this problem, we found clues indicating that the effects of enteral nutrition may be related to the severity of the ALI/ARDS. Finally, we did not assess the discrepancy of the ratio of partial pressure arterial oxygen and fraction of inspired oxygen (PaO2/FiO2 ratio) because of inadequate information. As one of the most frequently used indicators of oxygenation and respiratory function, the PaO2/FiO2 ratio is a good predictor of the condition of ALI/ARDS patients. Thus, further trials should report more information about it.

## 5. Conclusions

Overall, based on the existing data, the enteral immunomodulatory diet (omega-3 fatty acid, γ-linolenic acid and antioxidant supplementation) could not reduce the mortality of patients with ALI/ARDS and also could not extend the 28-day ventilator-free days or 28-day ICU-free days. However, the subgroup analysis showed that enteral immunomodulatory nutrition could benefit ALI/ARDS patients with high mortality, but it should be used with discretion. More well-designed clinical trials are urgently needed to verify this conclusion.

## Supplementary Information

Table S1: Studies excluded from the meta-analysis of clinical trials involving enteral nutrition treatment for ALI/ARDS.

Table S2: Risk of bias of the included studies.

Figure S1: Sensitivity results (overall effect when excluding the trial conducted by Rice T. *et al.*).

Figure S2: Funnel plot of the standard error by log relative risk of all-cause mortality.
