# Supplementary material for: Enteral Immunomodulatory Diet (Omega-3 Fatty Acid, γ-Linolenic Acid and Antioxidant Supplementation) for Acute Lung Injury and Acute Respiratory Distress Syndrome: An Updated Systematic Review and Meta-Analysis
Source: Nutrients. 2015 Jul 9;7(7):5572–85. doi: 10.3390/nu7075239 (PMC4517016; doi:10.3390/nu7075239)
Supplement: Supplementary file 1 [file nutrients-07-05239-s001.docx]

Supplementary Information

**Table S1.** Studies excluded from the meta-analysis of clinical trials involving enteral nutrition treatment for ALI/ARDS.

| **Study** | **Reason for Exclusion** |
| --- | --- |
| Nelson *et al.*, 2003 [1] | Did not report data about mortality |
| Pacht *et al.*, 2003 [2] | Included in other studies. |
| Theilla *et al.*, 2007 [3] | Did not report data about mortality. |
| Pontes-Arruda *et al.*, 2011 [4] | The patients did not meet the inclusion criteria. |
| Schott *et al.*, 2012 [5] | Repeated reports. |


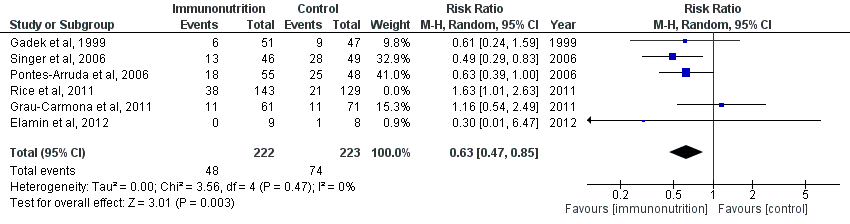
ALI, acute lung injury; ARDS, acute respiratory distress syndrome.

**Figure S1.** Sensitivity results (overall effect when excluding the trial conducted by Rice T. *et al*.).


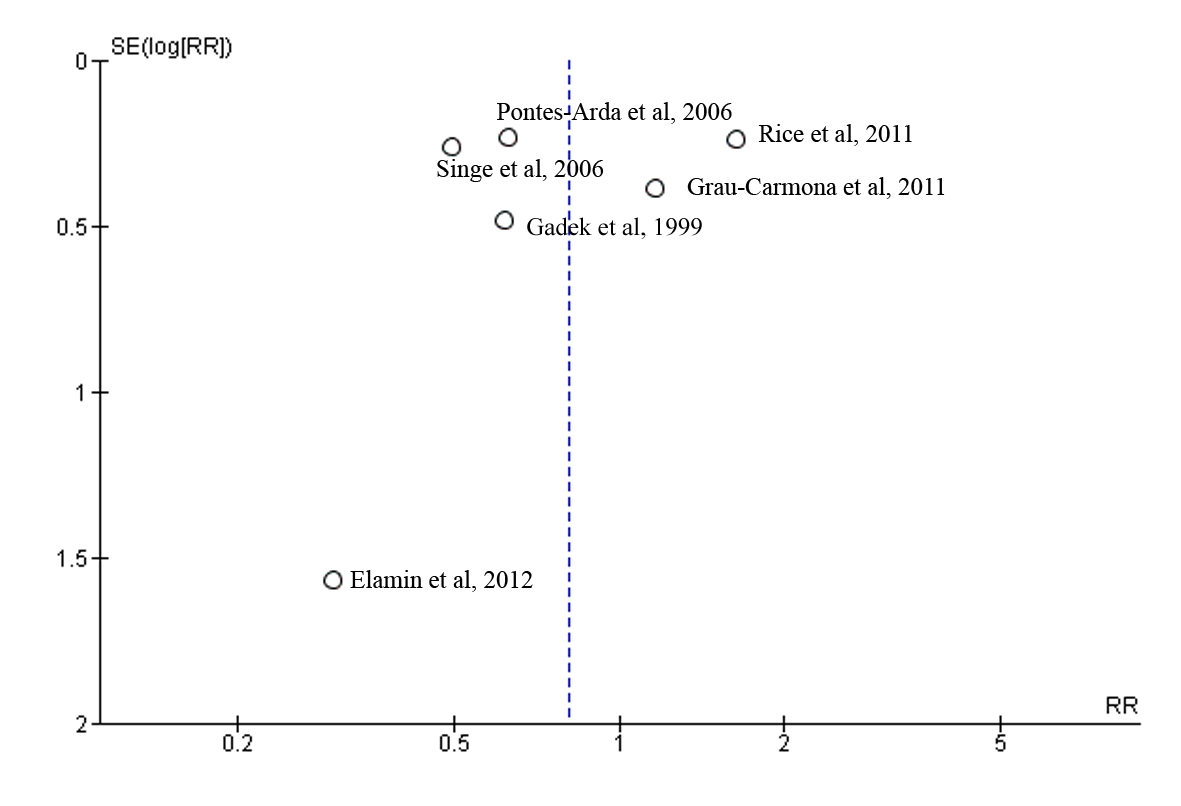


**Figure S2.** Funnel plot of the standard error by log relative risk of all-cause mortality.

**Table S2.** The risk of bias of the included studies.

| **Study** | **Selection Bias** | | **Performance Bias** | **Detection Bias** | **Attrition Bias** | **Reportion Bias** | **Other Bias** | **Modified Jadad Scale** |
| --- | --- | --- | --- | --- | --- | --- | --- | --- |
|  | **Random Sequence Generation** | **Allocation Concealment** | **Blinding  of Participants and Personnel** | **Blinding  of Outcome Assessment** | **Incomplete Outcome Data** | **Selective Reporting** | **Other Sources  of Bias** |  |
| Gadek *et al.*, 1999 [6] | Low risk | Low risk | Low risk | Low risk | Low risk | Low risk | High risk ^a^ | 7 |
| Singer *et al.*, 2006 [7] | Low risk | Low risk | High risk | High risk | Low risk | Low risk | Low risk | 5 |
| Pontes-Arruda *et al.*, 2006 [8] | Unclear | Unclear | Low risk | Low risk | Low risk | Low risk | High risk ^a^ | 5 |
| Grau-Carmona *et al.*, 2011 [9] | Low risk | Low risk | High risk | High risk | Low risk | Low risk | Low risk | 5 |
| Rice *et al.*, 2011 [10] | Low risk | Low risk | Low risk | Low risk | Low risk | Low risk | Low risk | 7 |
| Elamin *et al.*, 2012 [11] | High risk | Low risk | Low risk | Low risk | Low risk | Low risk | Low risk | 5 |

^a^ The results of this study might be affect by the large drop-out.

**References**

1. Nelson, J.L.; DeMichele, S.J.; Pacht, E.R.; Wennberg, A.K. Enteral Nutrition in ARDS Study Group. Effect of enteral feeding with eicosapentaenoic acid, gamma-linolenic acid, and antioxidants on antioxidant status in patients with acute respiratory distress syndrome. *JPEN J. Parenter.
   Enter. Nutr.* **2003**, *27*, 98–104.
2. Pacht, E.R.; DeMichele, S.J.; Nelson, J.L.; Hart, J.; Wennberg, A.K.; Gadek, J.E. Enteral nutrition with eicosapentaenoic acid, gamma-linolenic acid, and antioxidants reduces alveolar inflammatory mediators and protein influx in patients with acute respiratory distress syndrome. *Crit. Care Med.* **2003**, *31*, 491–500.
3. Theilla, M.; Singer, P.; Cohen, J.; Dekeyser, F. A diet enriched in eicosapentanoic acid,
   gamma-linolenic acid and antioxidants in the prevention of new pressure ulcer formation in critically ill patients with acute lung injury: A randomized, prospective, controlled study. *Clin. Nutr.* **2007**, *26*, 752–757.
4. Pontes-Arruda, A.; Martins, L.F.; de Lima, S.M.; Isola, A.M.; Toledo, D.; Rezende, E.; Maia, M.; Magnan, G.B.; Investigating Nutritional Therapy with EPA, GLA and Antioxidants Role in Sepsis Treatment (INTERSEPT) Study Group. Enteral nutrition with eicosapentaenoic acid, gamma-linolenic acid and antioxidants in the early treatment of sepsis: Results from a multicenter, prospective, randomized, double-blinded, controlled study: The INTERSEPT study. *Crit. Care* **2011**, *15*, R144.
5. Schott, C.K.; Huang, D.T. Omega-3 fatty acids, gamma-linolenic acid, and antioxidants: Immunomodulators or inert dietary supplements? *Crit. Care* **2012**, *16*, 325.
6. Gadek, J.E.; DeMichele, S.J.; Karlstad, M.D.; Pacht, E.R.; Donahoe, M.; Albertson, T.E.; Van Hoozen, C.; Wennberg, A.K.; Nelson, J.L.; Noursalehi, M. Effect of enteral feeding with eicosapentaenoic acid, gamma-linolenic acid, and antioxidants in patients with acute respiratory distress syndrome. *Crit. Care Med.* **1999**, *27*, 1409–1420.
7. Singer, P.; Theilla, M.; Fisher, H.; Gibstein, L.; Grozovski, E.; Cohen, J. Benefit of an enteral diet enriched with eicosapentaenoic acid and gamma-linolenic acid in ventilated patients with acute lung injury. *Crit. Care Med.* **2006**, *34*, 1033–1038.
8. Pontes-Arruda, A.; Aragao, A.M.; Albuquerque, J.D. Effects of enteral feeding with eicosapentaenoic acid, gamma-linolenic acid, and antioxidants in mechanically ventilated patients with severe sepsis and septic shock. *Crit. Care Med.* **2006**, *34*, 2325–2333.
9. Grau-Carmona, T.; Moran-Garcia, V.; Garcia-de-Lorenzo, A.; Heras-de-la-Calle, G.; Quesada-Bellver, B.; Lopez-Martinez, J.; Gonzalez-Fernandez, C.; Montejo-Gonzalez, J.C.; Blesa-Malpica, A.; Albert-Bonamusa, I.; *et al.* Effect of an enteral diet enriched with eicosapentaenoic acid, gamma-linolenic acid and anti-oxidants on the outcome of mechanically ventilated, critically ill, septic patients. *Clin. Nutr.* **2011**, *30*, 578–584.
10. Rice, T.W.; Wheeler, A.P.; Thompson, B.T.; DeBoisblanc, B.P.; Steingrub, J.; Rock, P. Enteral omega-3 fatty acid, gamma-linolenic acid, and antioxidant supplementation in acute lung injury. *JAMA* **2011**, *306*, 1574–1581.

1. Elamin, E.M.; Miller, A.C.; Ziad, S. Immune enteral nutrition can improve outcomes in medical-surgical patients with ARDS: A prospective randomized controlled trial. *J. Nutr. Disord. Ther.* **2012**, *2*, 109.

© 2015 by the authors; licensee MDPI, Basel, Switzerland. This article is an open access article distributed under the terms and conditions of the Creative Commons Attribution license (http://creativecommons.org/licenses/by/4.0/).
